# Cervical Cancer and Human Papillomavirus Awareness among Women in Antigua and Barbuda

**DOI:** 10.3390/medicina59071230

**Published:** 2023-06-30

**Authors:** Prasanna Honnavar, Edmond Mansoor, Cherie Tulloch, Uttam Udayan, Isabella Cosmello, Purva Patel, Ashley Bersma

**Affiliations:** 1 Department of Microbiology and Immunology, American University of Antigua College of Medicine, St. Johns 1451, Antigua and Barbuda; uudayan@auamed.net; 2 Department of Clinical Medicine, American University of Antigua College of Medicine, St. Johns 1451, Antigua and Barbuda; emansoor@auamed.net; 3 Department of Obstetrics and Gynecology, Sir Lester Bird Medical Centre, St. Johns 1451, Antigua and Barbuda; cherietulloch@gmail.com; 4 Basic Medical Science, American University of Antigua College of Medicine, St. Johns 1451, Antigua and Barbuda; isabellac@auamed.net (I.C.); purvap@auamed.net (P.P.); ashleyber@auamed.net (A.B.)

**Keywords:** cervical cancer, papillomavirus infections, HPV vaccine, HPV testing, Antigua and Barbuda, public health education

## Abstract

*Background and objectives*: Cervical cancer is the fourth leading cause of cancer-related deaths in women. Human papilloma virus (HPV) is known to cause cervical cancer. The incidence and mortality of cervical cancer has drastically reduced due to effective vaccination against HPV in developed countries. The projected rise in cervical cancer cases in Latin American and Caribbean countries necessitates a study to evaluate awareness about HPV, cervical cancer, the HPV vaccine, and prevention among women in Antigua and Barbuda. *Materials and methods*: This was an observational, cross-sectional study. The participants were women aged between 18 and 65 years, residing in Antigua and Barbuda. The study was conducted over the period of February to April 2023. After taking informed consent electronically, sociodemographic and behavioral data was collected through questionnaires sent out as links and QR-codes and were analyzed by Qualtrics^XM^. The association between the demographic groups and awareness about HPV, cervical cancer, the HPV vaccine, and prevention was analyzed by a Chi-square test. *Results*: In total, 467 women were included in the study. The percentage of participants aware of HPV was 91.6% (*n* = 412). A total of 70.7% (*n* = 318) and 56.7% (*n* = 255) women were aware that cervical cancer is caused by HPV and is sexually transmitted, respectively. Although 70.6% (*n* = 315) of participants were aware that the vaccine protects against HPV, only 12.8% (*n* = 57) were vaccinated. Of the participants, 49.7% (n = 192) were willing to get vaccinated. The percentage of participants aware of the Papanicolaou (Pap) smear procedure was 98.9% (*n* = 435) and 87.8% (*n* = 382) had a Pap smear within the last 10 years while 12.2% (*n* = 53) never had a Pap smear screening. The willingness to know more information about HPV and the HPV vaccine among the participants was 77% (*n* = 335). *Conclusions*: The overall awareness among women in Antigua and Barbuda about HPV, cervical cancer, the HPV vaccine, and prevention was high. We recommend a national health education program and vaccine drive to complement our findings.

## 1. Introduction

Every year, 690,000 cancer cases are attributed to Human Papilloma Virus (HPV) infection. According to the 2023 World Health Organization International Agency for Research on Cancer’s Global Cancer Observatory Report, incidence of cancer caused by HPV infection was 6.9, 9.2 and 16.1 per 100,000 women in high and upper-middle-income, lower-middle-income, and low-income countries, respectively [1]. Cervical cancer has been the third leading cause of death in women in Antigua and Barbuda according to national data from 2014 to 2018 [2].

Almost all cases of cervical cancer are caused by high-risk types of HPV and the resulting precancerous changes in the cervix [3,4]. HPV is sexually transmitted from person to person. According to global reports, 75% of sexually active individuals contract HPV over their lifespan [3]. Although more than 200 genotypes of HPV have been identified, a group of 15 high-risk types are considered as an essential factor for the development of cervical cancer as well as vulvar, vaginal, penile, and oropharyngeal cancers [5]. HPV 16 and 18 are considered etiologically determinant agents in over 71% of cervical cancer cases. The prevalence of specific HPV types varies geographically. Although the infection is normally transient, its persistence has been directly associated with the development of cervical cancer, which represents an important health problem due to its high incidence. 

Based on population growth and the projected burden, cervical cancer incidence in Latin America and the Caribbean is expected to climb 19–132% and HPV vaccinations have reduced the incidence and mortality of cervical cancer in higher-income countries by up to 80% [6,7]. The HPV vaccine was first approved by the US Food and Drug Administration in 2006 [8]. Two HPV vaccines are widely available at present, and a couple of vaccines are in the pipeline. When the prevalence of HPV increased in 2014, 84 countries included the HPV vaccine in their national vaccination and immunization programs [9]. 

The ministry of health, wellness, and environment (MOHWE) of Antigua and Barbuda introduced Gardasil (the quadrivalent HPV vaccine) as part of the national immunization schedule in 2018 [9]. The HPV vaccine is currently available throughout the island at all clinics free of charge [9]. A total of 15% of the target population of children aged 9–13 years received at least one dose of HPV vaccine in the first year of the program. However, the vaccination rate declined significantly during the COVID-19 pandemic. Gardasil 9 is also available on the island but only through private care with a minimal fee. 

In Antigua and Barbuda, a Papanicolaou (Pap) smear is recommended to all women from the age of 21 years whether sexually active or not. The target age group is 21 to 65 years. All Pap smears done at public health facilities are free of cost. About seven in ten women have been screened for cervical cancer in the last 5 years [2]. In Antigua and Barbuda, there is a scarcity of data on women’s awareness and comprehension of HPV. Research documenting HPV vaccine acceptability and cultural influences is a critical public health requirement in the Caribbean region. The major source of HPV awareness among the young population may be social media and some web blogs. No study has evaluated either the knowledge of Antiguan and Barbudan women about HPV or the awareness of cervical cancer prevention and screening. The purpose of this study was to analyze the awareness, knowledge, attitudes, and practices of women in Antigua and Barbuda regarding HPV, cervical cancer, the HPV vaccine, and prevention. This study’s findings are meant to give a baseline understanding of the risk of HPV infection, allowing public health officials to act if necessary. Studies have shown that social, cultural, and economic aspects are linked to the uptake of HPV vaccines throughout time. The results of this study can also help to fashion policies and inform decision makers in that they should be aware of the overall level of knowledge and implement educational interventions to address the information gap and assist health practitioners in consistently recommending the HPV vaccine.

## 2. Material and Methods

### 2.1. Study Design

This was an observational, cross-sectional, survey-based study conducted in Antigua and Barbuda during the period from February to April 2023. The Institutional Review Board, American University of Antigua College of Medicine (050922-1, 5 November 2022) and Institutional Review Board, Antigua and Barbuda Ministry of Health (AL-01/102022-ANUIRB, 24 October 2022) approved the study protocol (E-20-5069 on 8 September 2020). Informed consent was obtained from all the participants electronically.

### 2.2. Study Setting and Study Population

The eligibility criteria for participants were female citizens/residents aged between 18 and 65 years, residing in Antigua for at least six months or more, and who had given informed consent to participate in the study. Participants who identified themselves as males and those who did not completely fill out the questionnaires were excluded. 

In order to obtain a confidence level of 95%, that the real value is within 5% of the measured/surveyed value, at least 383 measurements or surveys were required [10]. However, 467 respondents participated in this study. The sample size was expanded to decrease the risk of sampling errors. To demonstrate the feasibility of the questionnaire, a pilot study with 25 women participants was carried out. According to the obtained results, appropriate changes were made.

### 2.3. Data Collection and Questionnaires

The survey was conducted using self-reported questionnaires prepared by a team of researchers and clinicians involved in this study. The study questionnaire contained 5 main sections with 23 close-ended questions (Supplementary File). Questionnaires were made available on Qualtrics^XM^ using a weblink and QR code [11]. The link and barcode were distributed using flyers, virtual social media platforms such as Instagram, Facebook, WhatsApp, people’s networks, and online news platforms such as Antigua Breaking News and Antigua Newsroom. The investigators visited and distributed flyers with the link to questionnaires in public places such as supermarkets and government offices, and to employees in hotels and resorts. The questionnaires were filled out voluntarily and anonymously. 

Section 1: Personal information

This section included 7 questions about general information such as gender (male; female), age in years (18 to 28; 29 to 38; 39 to 48; more than 59), marital status (single; married; living with partner; divorced; widowed), race or ethnicity (Afro-Caribbean; White-Caribbean; Indo-Caribbean; Hispanic), education level (last level completed: primary school; high school; some college; college graduate or higher), visits to health care providers in the last year (yes; no; don’t remember), and health insurance (yes; no; don’t know). 

Section 2: Awareness about cervical cancer and HPV:

This section included 5 questions about the participants’ knowledge and awareness of cervical cancer and HPV: what are the sources of cervical cancer information (online newspaper; print newspaper; special health or medical magazine; radio; local television; internet; social media; health centers and doctor’s office), have they heard about HPV (yes; no), do they know that cervical cancer is caused by HPV (yes; no; don’t know), is HPV sexually transmitted (yes; no; don’t know), and can HPV infect both men and women (yes; no; don’t know). 

Section 3: Awareness about HPV vaccine, willingness, and barriers to get vaccinated:

This section included 5 questions about the participants’ knowledge and awareness on the HPV vaccine’s existence (yes; no; don’t know), have they ever had HPV vaccination (yes; no), do friends/parents approve of HPV vaccination (approve; disapprove; don’t know), what are the barriers to getting HPV vaccination (religion; worried about side effects; accessibility; too expensive/no insurance/cost; doctor didn’t tell me about it; not required; laziness; I am over the eligible age group/more than 45 years), and willingness to take the HPV vaccine if offered (yes; no; don’t know). 

Section 4: Awareness about Pap smear, willingness, and barriers to get screened:

This section included 4 questions about the participants’ knowledge and awareness of cervical cancer screening tests: do they know what a Pap smear is (yes; no), when was the last Pap smear test done (never; within the last 6 to 10 years; within the last 4 to 5 years; within the last 3 years), why have they not had a Pap smear (too young; no reason/never thought about it; doctor didn’t tell me about it; not required; laziness; too expensive/no insurance/cost; too painful, unpleasant; embarrassing; don’t know where to get it done; not so easily available; working hours collide with testing time), and if women who received the HPV vaccine still required periodic Pap smears (true; false; don’t know). 

Section 5: Awareness about HPV prevention and need for more information on HPV and cervical cancer:

This section included 2 questions about the knowledge and awareness of HPV prevention: how to decrease the risk of getting HPV infection (avoiding sex; vaccination; using condoms; antibiotics; herbal medication; don’t know), and do they need more information on HPV and cervical cancer (agree; disagree; don’t know). 

### 2.4. Statistical Analysis

Categorical variables described by percentages and frequencies were used to summarize the responses to each question. To examine the relationship between two categorical variables, the Chi-square test was used. The association between demographic groups and informative factors and knowledge, awareness about HPV, cervical cancer, the HPV vaccine, the HPV screening test, and prevention was analyzed by the Chi-square test [10]. *p* values < 0.05 were considered statistically significant.

## 3. Results

Section 1: General information. Women responders belonged predominately to the age group of 29 to 58 years and were Afro-Caribbean in origin. Half of the participants (*n* = 233, 50%) were single. The majority (49.1%) of responders were college graduates or had higher education. The number of participants visiting health care providers within the last year was 369 (79.9%) and nearly half of them (51.9%) had health insurance. Sociodemographic characteristics of women included in the study are shown in Table 1. 

**Figure 1:**
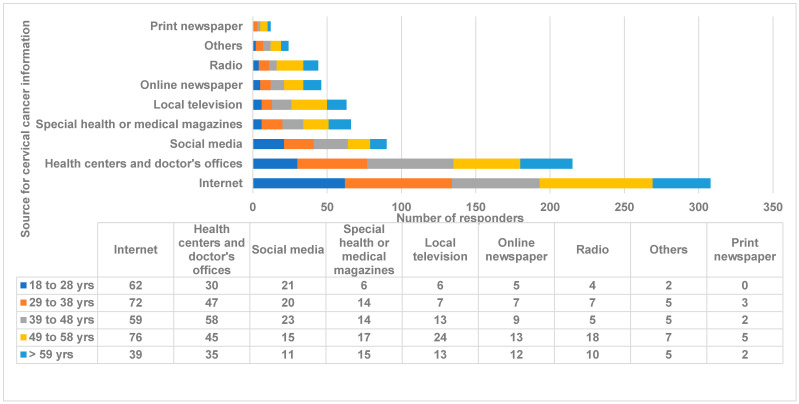
Age-wise distribution of sources of cervical cancer information. Comparison of the distribution of the source of cervical cancer information among women with different age groups. The *X*-axis represents the number of responders. The *Y*-axis represents the source of cervical cancer information from different age groups of women. The numbers indicate the number of responders. Antigua and Barbuda, 2023.

**Figure 2:**
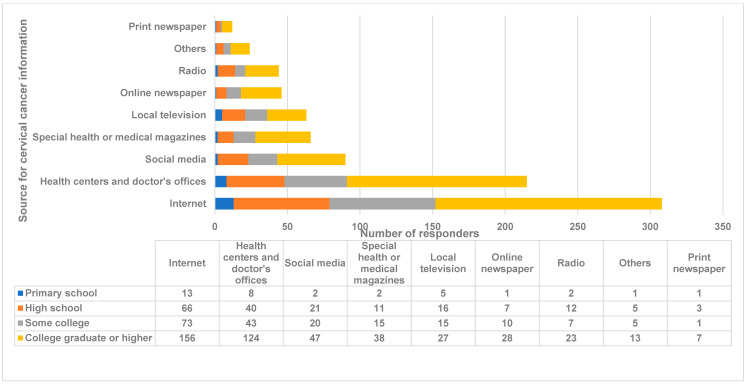
Education-wise distribution of sources of cervical cancer information. Comparison of the sources of cervical cancer information by education level. The *X*-axis represents the number of responders. The *Y*-axis represents the source of cervical cancer information from respondents by education level. The numbers indicate the number of responders. Antigua and Barbuda, 2023.

Section 2: Awareness of cervical cancer and HPV. The Internet (*n* = 308, 68.3%) and health centers/doctors’ offices (*n* = 215, 47.7%) were the major sources for getting cervical cancer information, followed by social media and health magazines (Figure 1). Women older than 49 years were significantly dependent on radio (*p* < 0.02) and television (*p* < 0.01) for getting the information, whereas the Internet was a major source of information for participants < 38 years (*p* < 0.0006) (Figure 1). Participants with higher education were significantly dependent on health centers/doctors’ offices for information (*p* < 0.0001) (Figure 2). A total of 412 (91.6%) participants had heard about HPV, and the majority of them had college-level/higher education (*p* < 0.001). The majority of responders who knew that HPV causes cervical cancer (*n* = 318, 70.7%) had a higher education level (*p* < 0.0004) (Figure 2). The higher-educated cohort was also significantly (*p* < 0.00001) more knowledgeable about the sexual transfer of HPV (*n* = 255, 56.7%). The majority of responders also knew that HPV could infect both men and women (*n* = 291, 64.8%). 

**Figure 3:**
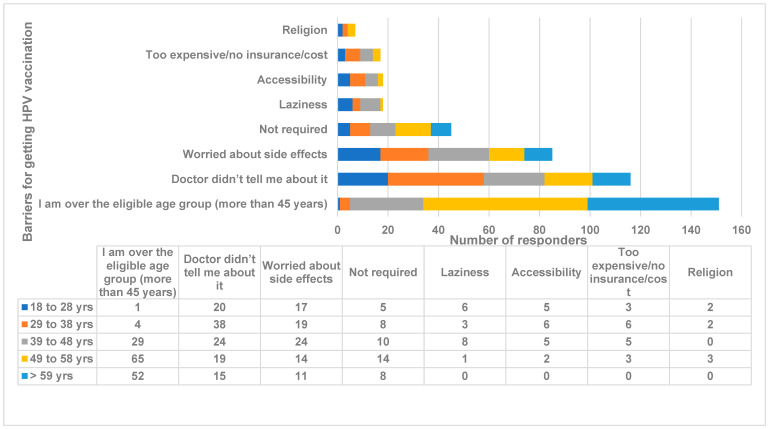
Different age groups of women’s barriers for getting HPV vaccination. Comparison of the different barriers to HPV vaccination reported by women with various age groups. The *X*-axis represents the number of responders. The *Y*-axis represents barriers for getting HPV vaccination from different age groups of women. The numbers indicate the number of responders. Antigua and Barbuda, 2023.

Section 3: Knowledge and awareness of the HPV vaccine. Most of the population knew that the vaccine could protect against HPV (*n* = 315, 70.6%) and, significantly, these were either college graduates or those with a higher education level (*p* < 0.00001). A significantly lower number of individuals was vaccinated against HPV (*n* = 57, 12.8%), and, significantly, they belonged to the 29 years and above age group (*p* < 0.00001). Most of the individuals (*n* = 389, 87.2%) did not get HPV vaccination. The reasoning for not getting vaccination was being in a higher age group (*n* = 151, 39.1%), no information being given by doctors (*n* = 116, 30.1%) and being concerned about the vaccine side effects. Other concerns were accessibility, laziness, cost, and religion. The 18 to 38 years age group was significantly worried about the side effects of the vaccine (*p* < 0.002) (Figure 3), accessibility (*p* < 0.02), no information from doctors (*p* < 0.0003) and laziness (*p* < 0.0006). Single women were significantly worried about vaccine side effects (*p* < 0.004) and no information was given by doctors (*p* < 0.007). Those who were living with a partner significantly thought that the vaccine was very expensive (*p* < 0.002). Indo-Caribbean and Hispanic women thought that the vaccine is not accessible and not required (*p* < 0.02). High school and college graduates thought that doctors should have informed them regarding the vaccine (*p* < 0.00001). Nearly half of the responders (18 to 48 years) showed a willingness to get vaccinated (*n* = 192, 49.7%) (*p* < 0.00001). 

Section 4: Knowledge and awareness on cervical cancer screening tests. Most of the population heard about Pap smears (*n* = 435, 98.9%). A total of 53 individuals never got screened by Pap smears. A significant number of them belonged to the age group less than 28 years (*p* < 0.00001) and were either single or a living with partner (*p* < 0.00001). The reason given was a too-young age (*n* = 16, 30.2%), never thought about it (*n* = 15, 28.3%) and laziness. The majority (*n* = 314, 72.5%) of them knew that women who received the HPV vaccine still required periodic Pap smears, and, significantly, they were the college-educated cohort (*p* < 0.00001). 

**Figure 4:**
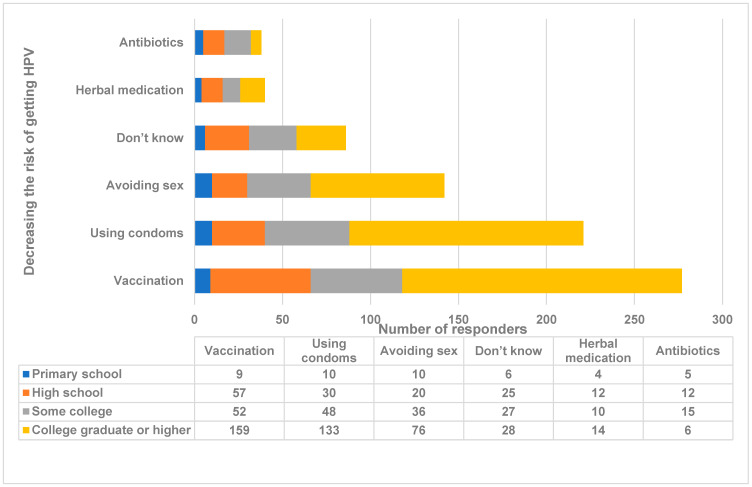
How to decrease the risk of getting HPV infection in comparison with education level. The distribution of knowledge regarding risk reduction strategies for HPV as stratified by education level. The *X*-axis represents the number of responders. The *Y*-axis represents knowledge of various HPV risk reduction strategies from the education level of respondents. The numbers indicate the number of responders. Antigua and Barbuda, 2023.

Section 5: Knowledge and awareness of HPV prevention. Most of participants (up to the 48-years age group) want to get more information about HPV and the vaccine (*n* = 335, 77%) (*p* < 0.001). The majority knew that the vaccine (*n* = 277, 64%) followed by condom usage (*n* = 221, 51%) can prevent HPV infection. A few individuals believed that herbal medication and antibiotics could prevent HPV infection. Higher education significantly correlated with knowledge of HPV prevention (*p* < 0.001) (Figure 4). Frequency and percentage of awareness of cervical cancer, HPV, the HPV vaccine, and screening test data are shown in Table 2. Table 3 shows the statistical relevance of sociodemographic characteristics with HPV awareness. Different sociodemographic variables (age, marital status, race, and education) were statistically compared with the awareness of participants on cervical cancer, HPV, the HPV vaccine, and cervical cancer screening tests. The comparison with *p* < 0.05 was considered statistically significant and is highlighted in Table 3. 

## 4. Discussion

To the best of our knowledge, this is the first study conducted in Antigua to assess the awareness of HPV/cervical cancer. The population was highly aware about cervical cancer, HPV, the HPV vaccine, Pap smears, and HPV prevention. Significant levels of the population also knew that HPV causes cervical cancer, HPV is transmitted by sexual intercourse, and that HPV can infect both men and women. The majority of the population also had the knowledge that vaccine and condom use can prevent HPV infection. The relatively high awareness was significantly due to a high education level, internet usage and robust health education provided at community and private clinics. The prevalence of HPV, insufficient preventive services, inadequate treatment access, socioeconomic conditions, certain cultural causes and values, and opinions about cervical cancer have been established as factors contributing to the occurrence of cervical cancer in women living in various parts of the world [12,13,14,15,16]. Due to the difficulty of implementing Pap smear screening programs and the lack of access to the HPV vaccine, the burden of cervical cancer remains disproportionately high in low- and middle-income countries, with more than 85% of worldwide cervical cancer deaths occurring in these regions.

The data from 2021 suggest that the total population of Antigua and Barbuda was approximately 93,220 [17]. There were 40.9 potentially passive people per 100 individuals. The potentially active people’s age group was between 15 and 64 years. There were 109.4 women per 100 men. Taking these data into consideration, there may be approximately 30,000 women in the age range of 18 to 64 years. 

Our results are in agreement with previous studies in which an international comparison was made on the knowledge of HPV and HPV vaccination [18]. However, among the few studies from the Caribbean region, it is a general perception that the awareness of cervical cancer/HPV, the HPV vaccine and HPV screening test is lacking among individuals from this part of the world [19]. The population under study, which is predominated by people of Afro-Caribbean origin, completely contradicts this. Similar to data from developed countries, a total of 91.6% individuals in Antigua knew about HPV [20,21]. Even some developed countries lack awareness of cervical cancer [22]. This percentage is significantly higher than studies from developing countries. [12,23,24]. There may be several factors that can contribute to differences in the awareness of cervical cancer between developed and developing countries. For example, healthcare access such as regular check-ups and screening tests such as Pap smears are typically higher in developed countries. Developed countries usually have higher literacy rates and better access to health education. Developed countries often have more resources to devote to health promotion campaigns. Women in developed countries, generally having higher incomes, may have more access to information and preventive healthcare services. In some developing countries, cultural beliefs and stigma surrounding sexual health can hinder the discussion of diseases such as cervical cancer, leading to reduced awareness. Policies supporting preventative healthcare, routine screenings, and vaccinations are implemented and enforced in developed countries.

In contrast to the younger age group, the older population was dependent on radio and television as their source of HPV information. Future national awareness programs can target social media and online media platforms. Participants with a higher education level gathered information from health centers. Although the population knew that the HPV vaccine can prevent the infection, only 12.8% were vaccinated. The main reasons for not getting the vaccination were concerns about vaccine side effects, accessibility, laziness, and religion. The younger age group was more worried about the vaccine’s side effects. However, half of the responders were willing to get vaccinated. Our study findings emphasize the need for a national policy of vaccinating more people. 

Most individuals had their Pap smears (77.8%), and only a few have not had the test because they thought that they belong to an age group that was too young, they never thought about it or laziness. Significant numbers of them belonged to the age group less than 28 years old and were single/living with a partner. The majority of those with a college education knew that women who received the HPV vaccine still required periodic Pap smears. Most of the responders wanted to get more information about HPV and its vaccine. The population knew that vaccine and condom usage could prevent HPV infection. A few individuals with a low-level education believed that herbal medication and antibiotics could prevent HPV infection. We recommend a national education program supported by public and private partners to enhance the easy availability of the HPV vaccine and HPV screening tests. 

The limitations of our study included the possibility of incorrect answers provided by responders to the online survey. There was a chance they may not be entirely truthful. There was a possibility that a few of the responders may have had gynecological diseases that led to hysterectomy. We were also unaware of their status on sexual activity (active or inactive). There may have been recall bias with respondents not remembering while answering the questions. The sample of women participating in the survey may not have been representative of all women in Antigua and Barbuda, as those who have access to smartphones or are well-versed in technology may have accessed information and answered the survey. Another limitation may have been the age group (18 to 65 years) involved in the study. Although Antigua and Barbuda has a predominately English-speaking population, other language (Spanish, French)-speaking groups may not have participated. As our study used a cross-sectional design, it could capture a snapshot of attitudes and awareness at a single point in time, but it could not track changes over time or determine causal relationships. Our questionnaire might not have covered all relevant factors related to the awareness and attitudes towards cervical cancer and HPV. As males were not included in the analyses, we do not know the awareness among men. It is a general perception that males’ awareness is lower than females’. A future study can target males. 

In conclusion, this was one of the first studies conducted in Antigua and Barbuda that showed that the awareness of HPV, cervical cancer, the HPV vaccine, and prevention was reasonably high. It can be compared to the level of awareness seen in some developed countries. Despite having high awareness, there was a low vaccination rate. The majority of responders wanted to get more information about HPV and its vaccine. We recommend a more robust National Health Education program on cervical cancer, HPV infection, and prevention, along with HPV vaccine drives in Antigua and Barbuda. 

## Figures and Tables

**Table 1:** Sociodemographic characteristics of women included in the study. Antigua and Barbuda, 2023.

Variables	Number of Responders	Category	Frequency (Total 464)	Percentage (%)
Age in years	467	18 to 28	76	16.3
		29 to 38	108	23.1
		39 to 48	101	21.6
		49 to 58	113	24.2
		>59	69	14.8
Marital status	466	Single	233	50
		Married	139	29.8
		Living with partner	40	8.6
		Divorced	34	7.3
		Widowed	9	1.9
		others	11	2.4
Race or ethnicity	466	Afro-Caribbean	395	84.8
		White-Caribbean	23	4.9
		Indo-Caribbean	7	1.5
		Hispanic	3	0.6
		Others	38	8.2
Education	464	Primary school	21	4.5
		High school	110	23.7
		Some college	105	22.6
		College graduate or higher	228	49.1
Visited health care provider in the last year	462	Yes	369	79.9
		No	85	18.4
		Don’t remember	8	1.7
Health insurance	462	Yes	240	51.9
		No	218	47.2
		Don’t remember	4	0.9

**Table 2:** Comparison of participants who had awareness of HPV infection, transmission, prevention of cervical cancer, the HPV vaccine with their source of information, willingness to get a Papanicolaou smear and get vaccinated, and barriers for vaccination. Antigua and Barbuda, 2023.

Variable	Number of Responders	Category	Frequency	Percentage (%)
Source of cervical cancer information	451	Online newspaper	46	10.2
		Print newspaper	12	2.7
		Special health or medical magazines	66	14.6
		Radio	44	9.8
		Local television	63	14
		Internet	308	68.3
		Social media	90	20
		Health centers and doctor’s offices	215	47.7
		Others	24	5.3
Heard of HPV	450	Yes	412	91.6
		No	38	8.4
HPV causes cervical cancer	450	Yes	318	70.7
		No	21	4.7
		Don’t know	111	24.7
HPV is sexually transmitted	450	Yes	255	56.7
		No	91	20.2
		Don’t know	104	23.1
HPV infects both men and women	449	Yes	291	64.8
		No	64	14.3
		Don’t know	94	20.9
The HPV vaccine protects against HPV	446	Yes	315	70.6
		No	28	6.3
		Don’t know	103	23.1
Vaccinated against HPV	446	Yes	57	12.8
		No	389	87.2
Parents/friends approve of you getting vaccinated against HPV	389	Approve	204	52.4
		Disapprove	16	4.1
		Don’t know	169	43.4
Barrier for getting the HPV vaccination	386	Religion	7	1.8
		Worried about side effects	85	22
		Accessibility	18	4.7
		Too expensive/no insurance/cost	17	4.4
		Doctor didn’t tell me about it	116	30.1
		Not required	45	11.7
		Laziness	18	4.7
		I am over the eligible age group (more than 45 years)	151	39.1
		Others	42	10.9
Willingness to get the HPV vaccine	386	Yes	192	49.7
		No	98	25.4
		Don’t know	96	24.9
Heard of the Pap smear	440	Yes	435	98.9
		No	5	1.1
When was the last Pap smear received	435	Never	53	12.2
		Within the last 6 to 10 years	58	13.3
		Within the last 4 to 5 years	47	10.8
		Within the last 3 years	277	63.7
Reason for not getting a Pap smear	53	Too young	16	30.2
		No reason/never thought about it	15	28.3
		Laziness	5	7.5
		Not required	4	9.4
		Too expensive/no insurance/cost	2	3.8
		Working hours collides with testing time	2	3.8
		Too painful, unpleasant	1	1.9
		Embarrassing	1	1.9
		Not so easily available	1	1.9
		Others	6	11.3
Women who received the HPV vaccine and still required periodic Pap smears	433	True	314	72.5
		False	15	3.5
		Don’t know	104	24
Would you like to get more information about HPV and the HPV vaccine	435	Agree	335	77
		Disagree	55	12.6
		Don’t know	45	10.3
How do you decrease the risk of getting HPV infection	433	Avoiding sex	142	32.8
		Vaccination	277	64
		Using condoms	221	51
		Antibiotics	38	8.8
		Herbal medication	40	9.2
		Don’t know	86	19.9

**Table 3:** Comparison of the statistical relevance of different sociodemographic variables with the awareness of participants. A total of 13 leading questions were analyzed and *p* < 0.05 was considered statistically significant. Antigua and Barbuda, 2023.

	Q1	Q2	Q3	Q4	Q5	Q6	Q7	Q8	Q9	Q10	Q11	Q12	Q13
Age in years	0.455	0.0814	0.188	0.626	0.272	<0.00001 *	0.743	<0.00001 *	0.0785	<0.00001 *	0.003 *	0.379	0.001 *
Marital status	0.164	<0.00001 *	0.202	0.0710	0.0563	0.0572	0.651	0.299	0.409	0.00001 *	0.261	0.397	0.507
Race or ethnicity	0.138	0.931	0.973	0.614	0.562	0.348	0.730	0.453	0.02 *	0.989	0.949	0.973	0.367
Education	0.001 *	0.0004 *	<0.00001 *	0.0779	<0.00001 *	0.0643	0.492	0.653	0.186	0.07	0.763	<0.00001 *	0.129

* *p* < 0.05 statistically significant. Q1—Heard about HPV; Q2—HPV causes cervical cancer; Q3—HPV is sexually transmitted; Q4—HPV infects both men and women; Q5—Is there a vaccine that protects against HPV; Q6—Have you been vaccinated against HPV; Q7—Friends/parents approve the HPV vaccine for you; Q8—Willingness to get the HPV vaccine; Q9—what is a Pap smear; Q10—When was your last Pap smear; Q11—Reason for not having a Pap smear; Q12—Women who received the HPV vaccine and still required periodic Pap smears; Q13—would you like to get more information about HPV and the HPV vaccine.

## Data Availability

The datasets used and/or analyzed as part of the present study are available from the corresponding author on reasonable request.

## Supplementary Materials

The following supporting information can be downloaded at https://www.mdpi.com/article/10.3390/medicina59071230/s1, Supplementary File: Default Question Block.
