# Supplementary material for: Cervical Cancer and Human Papillomavirus Awareness among Women in Antigua and Barbuda
Source: Medicina (Kaunas). 2023 Jun 30;59(7):1230. doi: 10.3390/medicina59071230 (PMC10383998; doi:10.3390/medicina59071230)
Supplement: Supplementary file 1 [file medicina-59-01230-s001.zip › medicina-2420389-supplementary.pdf]

Default Question Block

Research design: Transversal survey study

Our research aim is to explore the knowledge, behavior and perception of Antigua and Barbuda women regarding Human Papillomavirus (HPV) infection, HPV vaccination and cervical cancer.

This is an anonymous self-filled questionnaire, and data from this study will be used as aggregate data without the possibility of identification of individual subjects/participants.

The study is being conducted by the American University of Antigua College of Medicine (AUACOM). For further information or concerns about this survey, please contact Drs. Edmond Mansoor or Prasanna Honnavar, phonnavar@auamed.net, 484-8900 Extn:1175.

It takes about 3 minutes to complete.

Please answer the following questions honestly.

Block 13

Do you consent to participate in this study?

- ○ Yes
- ○ No

Block 1

The first group of questions are about yourself and your current status. There are no right or wrong answers.

Block 12

Gender

- ○ Male

- ○ Female

Block 2

Age in years

- ○ 18 to 28
- ○ 29 to 38
- ○ 39 to 48
- ○ 49 to 58
- ○ More than 59

Marital status

- ○ Single
- ○ Married
- ○ Living with partner
- ○ Divorced
- ○ Widowed
- ○ [ ] Others

Race or ethnicity

- ○ Afro-Caribbean
- ○ White-Caribbean
- ○ Indo-Caribbean
- ○ Hispanic
- ○ [ ] Others

Education (last level completed)

- ○ Primary school
- ○ High school
- ○ Some college
- ○ College graduate or higher

Have you seen your health care provider in the last year?

- ○ Yes
- ○ No
- ○ Don’t remember

Do you have health insurance?

- ○ Yes
- ○ No
- ○ Don’t know

Block 3

These next questions are about your knowledge and awareness of Human Papillomavirus. There are no right or wrong answers.

Block 4

Where do you look for cervical cancer information? (you can choose more than one option)

- ☐ Online newspaper
- ☐ Print newspaper
- ☐ Special health or medical magazines
- ☐ Radio
- ☐ Local television
- ☐ Internet
- ☐ Social media
- ☐ Health centers and doctor's offices
- ☐ ________ Others

Have you heard of Human Papillomavirus or HPV? (HPV is not the same as HIV)

- ○ Yes

- No

Does HPV cause cervical cancer?

- Yes
- No
- Don’t know

Is HPV sexually transmitted?

- Yes
- No
- Don’t know

Can HPV infect both men and women?

- Yes
- No
- Don’t know

Block 5

These next questions are about your knowledge and awareness of Human Papillomavirus vaccine. There are no right or wrong answers.

Block 6

Is there a vaccine that protects against HPV (also known as Gardasil)?

- Yes
- No
- Don’t know

Have you been vaccinated against HPV?

- Yes

- No

If your parents/friends knew about the HPV vaccine, would they approve of you getting vaccinated against HPV?

- Approve
- Disapprove
- Don't know

What is your barrier for getting the HPV vaccination? (you can choose more than one option)

- [ ] Religion
- [ ] Worried about side effects
- [ ] Accessibility
- [ ] Too expensive/no insurance/cost
- [ ] Doctor didn't tell me about it
- [ ] Not required
- [ ] Laziness
- [ ] I am over the eligible age group (more than 45 years)
- [ ] ________ Others

I am willing to get the HPV vaccine.

- Yes
- No
- Don't know

Block 7

These next questions are about your knowledge and awareness of cervical cancer testing. There are no right or wrong answers.

Block 8

Do you know what is a Pap smear?

- Yes
- No

When was your last Pap smear?

- Never
- Within the last 6 to 10 years
- Within the last 4 to 5 years
- Within the last 3 years

Why haven't you had a Pap smear? (ONE most important reason)

- Too young
- No reason/never thought about it
- Doctor didn't tell me about it
- Not required
- Laziness
- Too expensive/no insurance/cost
- Too painful, unpleasant
- Embarrassing
- Don't know where to get it done
- Not so easily available
- Working hours collides with testing time
- Others

Women who receive the HPV vaccine still require periodic Pap smears?

- True
- False
- Don't know

Block 9

The last questions are about your knowledge and awareness of Human Papillomavirus prevention

Block 10

I would like to get more information about HPV and the HPV vaccine.

- ○ Agree
- ○ Disagree
- ○ Don't know
- ○ [ ] If you agree, how?

How do you decrease the risk of getting HPV infection? (you can choose more than one option)

- ☐ Avoiding sex
- ☐ Vaccination
- ☐ Using condoms
- ☐ Antibiotics
- ☐ Herbal medication
- ☐ Don't know

Powered by Qualtrics
